# First Recorded Mating Flight of the Hypogeic Ant, *Acropyga epedana,* with its Obligate Mutualist Mealybug, *Rhizoecus colombiensis*

**DOI:** 10.1673/031.007.1101

**Published:** 2007-02-19

**Authors:** Chris R. Smith, Jan Oettler, Adam Kay, Carrie Deans

**Affiliations:** ^1^ Program in Ecology and Evolutionary Biology, University of Illinois Urbana-Champaign, USA; ^2^ Biologie 1, Universität Regensburg, 93040 Regensburg, Germany; ^3^ Department of Biology, University of St. Thomas, St. Paul, MN, USA

**Keywords:** *Rhizoecus*, mealybug, mutualism, trophobiosis, trophophoresy

## Abstract

On 26-July, 2005 a mating aggregation of Acropyga epedana Snelling (Hymenoptera: Formicidae) was observed in the Chiricahua Mountains in south-eastern Arizona. This is the first record of a mating flight of *A. epedana,* the only nearctic member of this pantropical genus. Mating behavior was observed, newly mated queens were collected, and a complete colony was excavated. New information is reported on the natural history and mating behavior of the species. The identity of a mealybug mutualist, *Rhizoecus colombiensis* (Homoptera: Rhizoecinae) is confirmed. Reproductive females participating in flights all carried mealybugs between their mandibles, indicating a vertical transfer of mealybugs with their ant hosts. No captured foundresses survived long in captivity, most likely due to the death of their mealybugs. The colony excavated had a single queen, though polygyny is common in the genus. Nearly all workers within the nest were heavily parasitized by mites, although males or gynes were not parasitized. These natural history observations are discussed with regard to this poorly understood mutualistic relationship between *Acropyga* ants and their mealybug partners.

## Introduction

The entirely hypogaeic and pan-tropical ant genus *Acropyga* Roger (Hymenoptera: Formicidae) lives in obligate symbiosis with mealybugs (Homopotera: Sterrnorhyncha: Coccoidea: Rhizoecinae). The ants feed on the sugar-rich feces of mealybugs which in turn feed on root-sap (Johnson et al. 2001), an association described as trophobiosis (Delabie 2001). Female mealybugs are carried by virgin queens during their nuptial flights (Johnson et al. 2001), a behavior called trophophoresy (LaPolla et al. 2002). Though workers, males, and queens of *A. epedana* Snelling have been previously collected and described in the Chiricahua Mountains in Arizona (LaPolla 2002), they seem rare, though this may be due to their underground lifestyle, and little is known about the species. This is the only species in the genus reported for the nearctic (LaPolla 2004), and it has been found only in southeastern Arizona. Here, we describe a mating flight of the species along with observations of colony founding, worker behavior, and parasitization of workers by mites.

## Results and Discussion

The mating aggregation and the colony that was sampled occurred in a shallow ditch alongside a primitive asphalt road, bordered by short grass, just 200 m west of the Southwestern Research Station (1645 m N31°53.028′ W109°12.378′), near Portal, Arizona. Beyond the ditch was a steep incline without vegetation and the ditch and road received direct sunlight for most of the day. The surrounding habitat was dominated by an open oak-pine-juniper forest.

**Figure 1.**
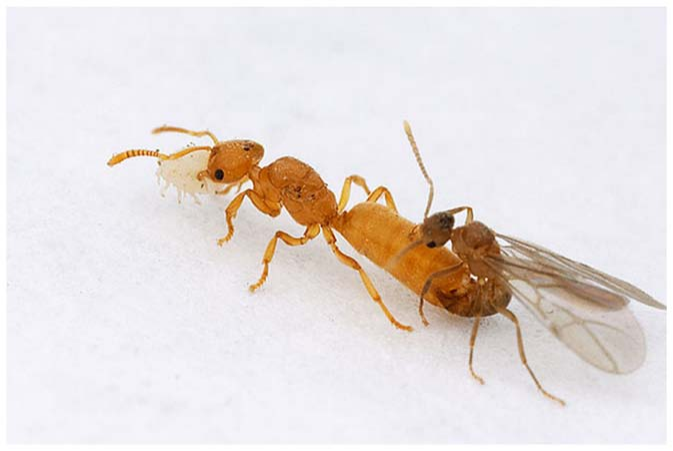
*Acropyga epedana* queen with mealybug between her mandibles and in copula with male. Photograph © Alex Wild 2005 (used with permission). This pair was collected during copulation when the female was alated. After the initial copulation was finished the queen dealated, and when photographs were taken the pair resumed copulation.

The mating flight occurred 2 days after the first heavy summer monsoon rains (26 July 2005), although there was very little precipitation on the day of the flight. The aggregation was first seen at ∼16:30h. Most of the males were hovering or swarming above small clumps of grass and rocks. Females entered the aggregation, after which multiple males simultaneously attempted to mate with them. Males out numbered females by ∼10:1. After mating most females immediately shed their wings and searched for suitable nest sites; only a few females flew from the aggregation. All females carried a mealybug in their mandibles. On 6 August 2005 at the same site, a single alate female carrying a mealybug was found actively mating, but no swarm could be found and only a single male was present (Figure 1).

Nine dealated queens were collected immediately after the nuptial flight on 26 July, and an additional 15 queens the next day under rocks at the same site. We transferred them into standard water tubes used for raising a colony from a foundress (a 20ml culture tube with ∼15ml water in the bottom stoppered with cotton, and sealed at the top with additional cotton). The 9 foundresses collected on the day of the nuptial flight lived for 36 ± 2 days (mean ± 1 SE). Of the 15 foundresses collected the day after the flight, only 4 were still alive by 31 July 2005 when they were transferred to a set of water tubes with live roots. Two remained alive through 6 August, and the final two died on the 8th and 30th of September. Another *A. epedana* dealate queen was collected under a rock on 2 August 2005, 4 km away from the original site (1839 m, N31°52.682′; W109°14.165′), and died on 10 September. None of the foundresses ever produced eggs.

A complete nest was excavated on 31 July 2005, a few meters from where the mating aggregation occurred. It was excavated to a depth of ∼30cm. The nest had a diameter of ∼30cm, and was partially beneath the asphalt road. To ensure the completeness of the excavation the hole was expanded in all directions for at least 5 cm after ants were last found. The nest consisted of a diffuse connection of small chambers and tunnels. At 20 cm depth elongate chambers were found along small roots of unknown species of plants. Mealybugs were on top of these roots and ants were nearby in the chamber. No ants or mealybugs were found in the upper grass roots. One *Acropyga* queen was collected from the nest at mid depth.

**Figure 2.**
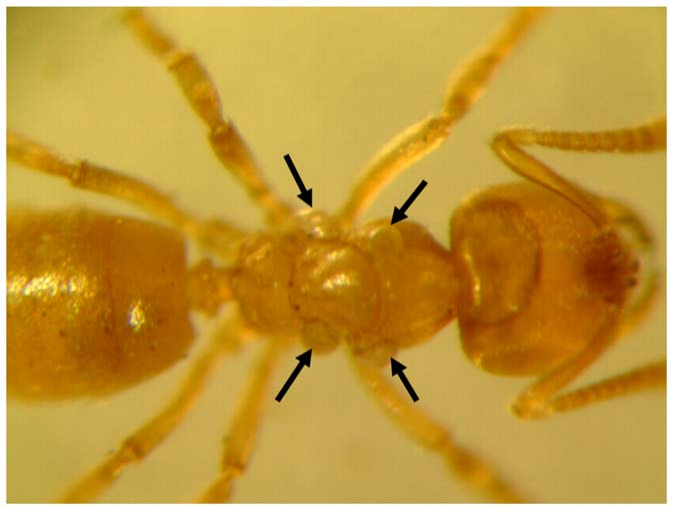
*Acropyga epedana* worker with mites, arrows point to mites. Photograph by C.R. Smith and J. Oettler.

The colony contained 234 workers, 1 queen, 48 mealybugs and 100 males. The presence of males indicates that the mating season was still in progress. Neither larvae nor female sexual offspring were found in the colony; only a single pupa was found, but it was too damaged to ascertain its caste. Upon arrival in the lab, the queen was attacked by several workers that repeatedly bit her and had a firm grasp of her legs. For observation, the colony was transferred into a plaster nest with grass roots inserted into chambers. The colony did not move into the protected chambers but stayed with the small pile of dirt they were introduced with, piling the mealybugs together on the dirt.

The majority of workers were found dead upon arrival at the lab. Workers exposed to microscope light immediately became agitated and quivered. Along with this photophobic behaviour the ants appeared to desiccate quickly. Approximately 95% of the workers were parasitized by small mites. The mites infested most body parts, but seemed to prefer the meso- and metathorax, with many workers having mites symmetrically positioned on either side of the thorax (Figure 2). Mites were also common on the underside of the head. Though most workers had 1–3 mites and some had up to 6, they did not seem to be physically impaired. After the death of an ant the mites immediately (within 1 min) detached and crawled away.

Three *Hypoponera inexorata* (Formicidae, Ponerinae) queen ants were found associated with *A. epedana* colonies. One *H. inexorata* queen with workers was found under the rock at the surface of the colony excavated on 31 July. Another was found under a rock with a founding *A. epedana* queen the day after the nuptial flight on 26 July. A third was found with *A. epedana* workers under a separate rock on the same day. The *H. inexorata* queen collected near the *A. epedana* colony was transferred to the plaster nest along with the colony. Though no aggression was noticed between the Hypoponera queen and *A. epedana* workers, the queen was found dead the following day.

Mealybugs collected with queens during flights and found within the colony were all *Rhizoecus colombiensis* (Homoptera: Rhizoecinae). Williams and LaPolla (2004) also collected this species from *A. epedana,* although this mealybug was previously only known from Colombia. The obligate mutualism between *Acropyga* ants and mealybugs is an excellent system for the study of co-evolution, including vertical transmission of both partners through time. Although an obligate association is apparent for both partners, across the single genus *Acropyga* there are several genera of mealybug (LaPolla 2002), indicating the possibility of multiple “domestication” events.

These observations on the mating flights and mating behavior of *A.* *epedana* are consistent with reports for other *Acropyga* species (Eberhard 1978; Buschinger et al. 1987; Williams 1998; Johnson et al. 2001). On the day that the mating swarm *of A. epedana* was observed, many other ant species also flew, likely cued by the beginning of the monsoon rains. The finding of *H. inexorata* queens in close association with *A. epedana* is most likely due to their similarity in flight time and preferential habitat under rocks.

The structure of the excavated nest was consistent with that reported previously for *A. epedana,* and for other *Acropyga* species. The size of the colony excavated, however, was an order of magnitude smaller than that reported for neotropical species (LaPolla et al. 2002). Only one queen was found in the nest though polygyny is suspected for this and other species, possibly as an adaptation to increase the genetic diversity of both the worker and mealybug populations within the nest (LaPolla et al. 2002).

The high parasitization rate of workers by mites is consistent with previous collections of *A.* *epedana* (LaPolla, personal communication). The cuticle of workers is very thin and light in color (LaPolla 2004) most likely a result of their subterranean lifestyle, which may increase their susceptibility to ectoparasites. On the other hand, the males and reproductive females that emerge from underground for nuptial flights were not parasitized, and survived in captivity for up to 6 weeks. This contrast may be due to physiological differences between the worker and reproductive castes, and the adaptations necessary for dispersal in this species. The eyes of workers were highly reduced in size relative to queens, which may affect their ability to migrate above ground.
